# Efficacy of Micromobile Foot Compression Device in Increasing Lower Limb Venous Blood Flow

**DOI:** 10.1155/2013/948769

**Published:** 2013-11-11

**Authors:** Thomas Charles, Stephen Mackintosh, James Fingleton, Irene Braithwaite, Mark Weatherall, Richard Beasley

**Affiliations:** ^1^Medical Research Institute of New Zealand, Private Bag Box 7902, Wellington 6242, New Zealand; ^2^Pacific Radiology Ltd., Wellington 5010, New Zealand; ^3^Capital & Coast District Health Board, Wellington 6242, New Zealand; ^4^University of Otago Wellington, Wellington 6242, New Zealand

## Abstract

*Background*. A novel, micromobile foot compression device (MMC) has been developed to reduce the risk of venous thromboembolism associated with prolonged seated immobility. *Objective*. To compare the efficacy of the MMC with graduated compression stockings in augmenting lower limb venous blood flow. *Patients/Methods*. Twenty participants were randomised to wear the MMC or a graduated compression stocking (GCS) on either the left or right leg while seated. Doppler ultrasound measurements of popliteal vein blood flow and leg circumference measurements were made −30 and −10 minutes (baseline) and +30 and +60 minutes following application of the interventions. The primary outcome variable was peak systolic velocity. A mixed linear model was used, with covariates including baseline measurement, randomised side, time, and a time by interaction term. *Results*. The mean popliteal vein peak systolic velocity at 60 minutes with the MMC was 20.1 cm/s which was significantly higher than with the GCS (difference 14.1 cm/s 95% CI 12.1–16.2), representing a 3.8-fold increase from baseline. *Conclusion*. The MMC resulted in a marked increase in lower limb venous blood flow which suggests that it may have efficacy in reducing the risk of venous thromboembolism associated with prolonged seated immobility, such as long distance air travel.

## 1. Introduction

Venous thromboembolism, encompassing both deep vein thrombosis (DVT) and pulmonary embolism, is a common condition associated with significant morbidity and mortality [1]. While the risk of venous thromboembolism in hospital inpatients is well established, it is only relatively recently that the potential role of prolonged seated immobility in the community has been recognised. Prolonged seated immobility, which can occur in a number of different situations including long distance air, car, and train travel, work, and recreation, has now been identified as one of the most important risk factors for venous thromboembolism [2–10]. For example, in the recent New Zealand case control study international air travel was associated with a 28-fold increased risk of venous thromboembolism, whereas prolonged work and computer related seated immobility was associated with a 2.8-fold increased risk [9].

This has led to consideration of the use of preventive measures in the community setting, with substantive efficacy having been demonstrated with graduated compression stockings (GCS) in the situation of prolonged air travel [11–13]. Another prophylactic measure recommended for in-hospital venous thromboembolism prevention is the use of intermittent calf or foot pneumatic compression devices [1]; however, major practical limitations prevent their implementation in at-risk individuals in the community. These limitations include difficulty in applying and removing the equipment, restrictions that the devices impose on clothing and ambulation, and the cost. Recently a novel micromobile foot compression device (MMC) has been developed, being potentially suitable for venous thromboembolism prevention in the ambulatory community setting, as it is portable, easy to wear, and comfortable to use [14]. Similar to other foot intermittent pneumatic compression devices, the MMC works by compressing the plantar venous plexus, thereby enhancing the physiological venous pump of the foot. The novel MMC has recently been shown to achieve a similar increase in lower limb venous blood flow, a validated IPC device commonly used in hospital practice [14]. The aim of our study was to compare the effect of the novel MMC with that of Class 1 GCS on lower limb venous blood flow during a period of sustained seated inactivity. Our hypothesis was that the MMC would lead to a greater increase in popliteal vein peak systolic velocity, mean flow velocity, and total volume flow than the GCS, whereas the GCS would lead to a greater reduction in vein diameter and leg circumference.

## 2. Methods

Healthy participants were recruited using a local volunteer database and word of mouth. Eligible participants met the following criteria: (1) aged between 18 and 65; (2) able to provide informed consent; (3) no history of current or previous DVT; (4) not currently pregnant; (5) no history or clinical features of peripheral vascular disease, peripheral neuropathy, scleroderma, lymphoedema, or joint deformity.

After an initial phone or email contact in April 2012, interested volunteers attended a screening visit at which written informed consent was obtained. Demographic and anthropometric measurements including height, weight, and body mass index (BMI) were recorded. A physical examination of the lower limbs and a brief medical history were conducted. Eligible subjects were then measured to enable correct sizing of the GCS to be used in the study. Twenty volunteers were screened, all of whom were eligible to participate. 

Participants attended an ultrasound clinic in May 2012. They were requested not to have undertaken any strenuous physical exercise, such as running or cycling, in the 24 hours preceding the ultrasound examination. Light exercise, such as walking or gentle swimming, was considered acceptable. Participants were required to wear shorts or a skirt for the measurements, without any leg coverings, to enable the ultrasonographer sufficient access to the popliteal fossa for probe positioning. Participants were seated upright in lightly padded straight backed chairs, with their legs relaxed and feet, without socks, elevated until the thigh was horizontal to the floor, so as to minimise any venous impairment caused by compression of the seat edge on the back of the thighs. An angle of 120 degrees between the femur and the tibia of both legs was achieved using a goniometer to standardise the degree of flexion between participants. 

The MMC was fitted to the left or right leg in accordance with a computer generated random allocation supplied by a biostatistician, and the GCS was then fitted to the same participant's opposite leg. Study staff assisted with the application of the interventions so as to minimise any leg muscle involvement. The MMC has a thick sole, so the opposite foot was raised to the same height by resting it on a dense foam platform of the same thickness. All prototype MMCs were large in size with adjustable straps to secure it to the foot. The MMC was positioned so that the midarch of the foot was directly above the pressure pad of the device.

Ultrasound measurements were performed at −30 and −10 minutes (baseline) before and then at +30 and +60 minutes following the application of the study interventions. Ultrasound measurements were performed in duplicate on both legs and the mean of the two readings determined the result at each time point. During the ultrasound measurements participants were instructed to remain very still and keep their leg and foot muscles relaxed and were encouraged to maintain relaxed regular breathing and refrain from talking. Calf and ankle circumference was measured after the ultrasound recordings at each time point. 

The ultrasound measurements were performed by an experienced sonographer using a Philips iU22 (Philips Medical Systems, Bothell, WA, USA, 2010) duplex ultrasound device with a variable high frequency linear array transducer (9–3 MHz). The angle of insonation was standardised at 60 degrees to the popliteal vein at the level of the crease in the popliteal fossa. A variable Doppler sampling gate was used to accurately capture venous volume flow.

Following completion of the final measurement participants completed a questionnaire which incorporated questions about their subjective experience of the interventions.

### 2.1. GCS

The GCS used in this study were custom-fitted, open-toed Class 1 (18–21 mmHg) “Mediven Plus A-D” below-knee graduated compression stockings (Medi GmbH & Co. KG, Medicusstraße 1, 95448 Bayreuth, Germany) which complied with the RAL-GZ 87 European standards (Hohenstein Research Institute, Schloßsteige 1, 74357 Bönnigheim, Germany).

### 2.2. MMC

**Figure 1:**
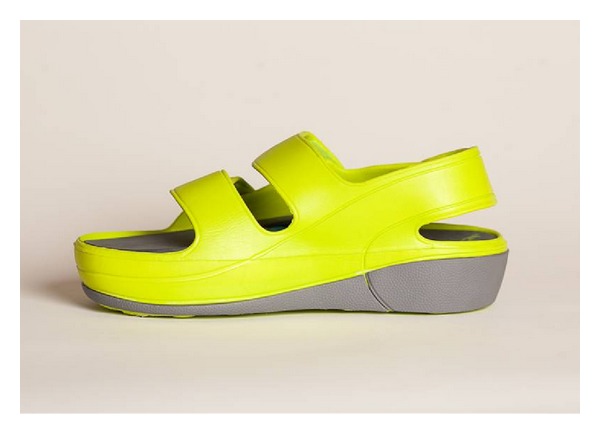
The MMC sandal which houses the compression device within the sole.

**Figure 2:**
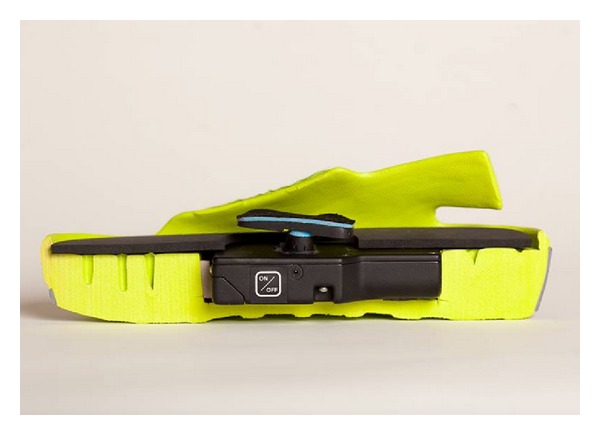
A lateral cross-section of the MMC sandal showing the embedded compression device in the sole. The pressure pad is in the elevated position.

**Figure 3:**
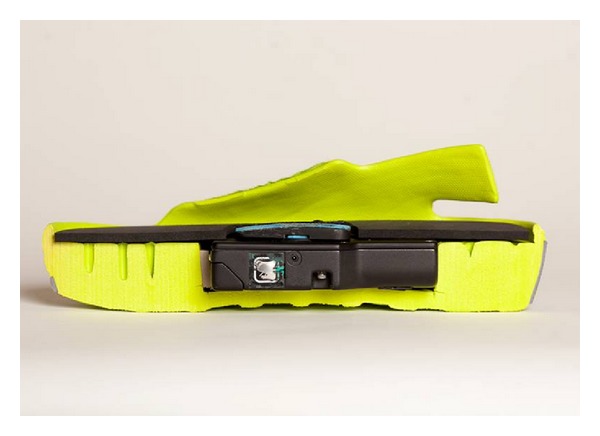
A lateral cross-section of the MMC sandal showing the embedded compression device in the sole. The pressure pad is in the retracted position.

The MMC (AVEX LLC, Grand Junction, CO, USA) consists of a rechargeable motorised thrusting arm and pressure pad beneath an inner sole of a purpose built sandal, which is fitted to the user with adjustable Velcro straps (Figure 1). The unit is user-activated by depressing a power switch on the body of the motor which is housed in a hollow within the sole of the sandal. Upon activation the device thrusts an 18.6 cm^2^ pressure pad directly onto the sole of the foot, compressing the plantar venous plexus (Figure 2). The MMC adjusts the height of its thrust until the exerted pressure on the arch of the foot reaches a measured 3.76 Newtons of pressure per cm^2^. The thrusting pad remains pressed on the arch of the foot for 2 seconds before retreating to its resting position (Figure 3). This thrust-pause-retreat cycle repeats every 20 seconds, while the wearer is stationary. A pressure sensitive interrupt switch is activated if the wearer stands up, so as not to deliver a thrust when the wearer is weight bearing. The device returns to its cyclical thrusting rhythm after 60 seconds of wearer inactivity.

### 2.3. Statistical Methods

The primary outcome variable was the peak systolic velocity (cm/s) in the popliteal vein at 30 and 60 minutes. Secondary outcome variables were the mean flow velocity (cm/s), total volume flow (L/sec), vein cross-sectional area (cm^2^), and the ankle and calf circumference at 30 and 60 minutes.

For the outcome measures, a mixed linear model was used, with covariates including the baseline measurement, a term for the randomisation (right versus left leg), time (two measurements at 30 and 60 minutes), and a time by treatment interaction term. The time by treatment interaction term tests if the difference between treatments is of the same magnitude at 30 minutes and 60 minutes. If this term is not statistically significant, then the main effect of treatment is the difference between treatments averaged over the two measurement times. The analysis used, adjusting for baseline (as a covariate), gives narrower confidence intervals than a change from baseline analysis. Participants were treated as a random effect to take account of the correlation of the measurements on the legs of the same participant.

### 2.4. Power Calculation

A pilot study demonstrated a standard deviation for paired differences in popliteal vein peak systolic velocity of 1.3 cm/s, and the mean peak systolic velocity in the seated position was around 3.5 cm/s [15]. A difference of 20% was considered to represent a significant difference in blood flow. A sample size of 20 participants had 80% power to detect a difference of 0.9 cm/s of peak systolic velocity, using a two-sided paired *t*-test at the 0.05 significance level.

The study was approved by the multiregion ethics committee (details) and registered with Australia and New Zealand Clinical Trials Registry (ACTRN12612000495820).

## 3. Results

The study was completed in 19 subjects, as one subject withdrew during the study due to syncope associated with hearing their blood flow through the ultrasound machine. The characteristics of the participants are shown in Table 1.

### 3.1. Peak Systolic Velocity

**Figure 4:**
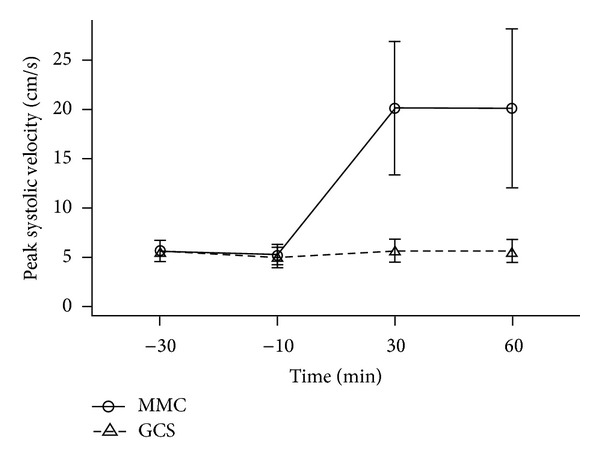
Peak systolic velocity measured in the popliteal vein at 30 and 10 minutes prior to the application of the GCS and MMC interventions and 30 and 60 minutes following their application.

The MMC caused a marked increase in peak systolic velocity (Table 2), which was significantly greater than with the GCS (difference 14.1 cm/s, 95% CI 12.1–16.2, *P* < 0.001) (Figure 4). For the MMC the mean (SD) peak systolic velocity at 60 min was 20.1 (8.1) cm/s, representing a 3.8-fold increase from baseline (Table 2). The mean (SD) peak systolic velocity at 60 minutes for the GCS was 5.6 (1.1) cm/s, which was minimally increased from baseline.

### 3.2. Secondary Outcome Variables

The MMC caused a marked increase in mean flow velocity and total volume flow (Table 2) which was significantly greater than with the GCS (difference 3.42 cm/s, 95% CI 2.87–3.98, *P* < 0.001 and 0.19 l/min, 95% CI 0.16–0.23, *P* < 0.001, resp.). The MMC resulted in a 2.9- and 3.0-fold increase from baseline, respectively, in these measures, whereas the GCS resulted in minimal change from baseline (Table 2).

There was no significant difference between MMC and GCS in vein cross-sectional area with no significant change from baseline with either device (difference 0.02 cm^2^, 95% CI −0.026–0.074, *P* = 0.34). The GCS resulted in a 1.2 cm smaller calf circumference compared with the MMC (95% CI 1.0–1.4, *P* < 0.001. There was no significant difference in ankle circumference between the two devices (difference −0.03 cm^2^, 95% CI −0.17–0.12, *P* = 0.72).

Subjective feedback from a questionnaire administered to the participants immediately following the final clinical measurement indicated that the MMC was considered both comfortable and easy to fit. Participants rated that they were highly likely to consider using the MMC in the hospital setting and may use it during air travel but less likely to use it seated at home or at work (Table 3).

## 4. Discussion

This study has shown that a novel, wearable, foot compression device (MMC) caused a marked increase in lower limb blood flow compared with the graduated compression stockings (GCS) in a model of seated immobility in healthy subjects. This performance suggests that the MMC may be effective in the prevention of venous thromboembolism associated with prolonged seated immobility, in which venous stasis is the predominant pathogenic mechanism.

The MMC resulted in 2.9- to 3.8-fold increases from baseline in popliteal vein peak systolic velocity, mean flow velocity, and total flow volume. These findings provide independent confirmation of the effects of the MMC demonstrated in the previous study, in which the MMC augmented venous velocity in the popliteal vein by 3.2-fold [14]. The magnitude of the MMC's effect on venous haemodynamics was also similar to that of the A-V Impulse System IPC device. The clinical relevance of these comparable effects is that the A-V Impulse System device has been shown to be effective in reducing the risk of venous thromboembolism after surgery [16–19]. In a randomised controlled trial of the A-V Impulse System after total knee replacement, major DVT occurred in only 17.8% of the intervention group compared with 59.4% of the control group [16]. Similarly, trials of the A-V Impulse System after total hip replacement have demonstrated a significant reduction in proximal and major calf thrombosis [17] with a benefit that is in addition to protection provided by subcutaneous heparin [18]. Prophylaxis using the A-V Impulse System has also been shown to significantly reduce the incidence of pulmonary embolism after hip replacement [19]. The comparable haemodynamic effects produced by the MMC and A-V Impulse System suggest that the MMC may have efficacy as a prophylactic measure to reduce the risk of venous thromboembolism secondary to orthopaedic surgery and potentially in other situations of prolonged immobility, such as long distance travel.

The apparent lack of a significant change from baseline in measures of popliteal vein blood flow or vein diameter with the GCS intervention does not necessarily indicate a lack of physiological effect, as blood flow progressively decreases and vein diameter increases over time with sitting [20]. It is probable that the GCS prevented these physiological changes that would have occurred in the model of venous stasis secondary to prolonged seated immobility. The GCS did prevent the increase in calf circumference which occurred with prolonged sitting with the MMC.

There are a number of methodological issues relevant to the interpretation of the study findings. Doppler ultrasound was employed as a highly sensitive, specific, and reproducible noninvasive method of measuring lower limb blood flow [21, 22]. Peak systolic velocity was chosen as the primary outcome variable as it represents the most consistent nonartefactual wave form detected by ultrasound. It was not possible to blind the sonographer to the intervention allocation, due to the requirement to undertake the ultrasound examination of popliteal vein close to the upper end of the stocking and this may have biased the results. Participants were instructed not to move their legs throughout the duration of clinical measurements to provide an accurate stable baseline measurement and to ensure that the findings related to seated immobility. While every effort was made to ensure that the participants kept their legs relaxed, it cannot be guaranteed that this was achieved at all times. Although it has been demonstrated that the right and left legs have similar venous haemodynamics [23], participants had the interventions randomised to either the right and/or left leg to avoid any potential difference. We studied healthy participants without vascular disease to minimise risk to the participant, to achieve accurate ultrasound measurements, and to ensure that our findings were generalisable to the healthy worker. 

In considering the potential utility of MMC in the community it is worthwhile reviewing the characteristics of venous thromboembolism resulting from prolonged seated immobility. In the case of air travel, this is a common risk factor worldwide with approximately 2 billion air travellers annually [24]. In the recent New Zealand case control study, air travel of at least 4-hour duration within four weeks of the index event occurred in 15% of patients presenting to hospital with a DVT or pulmonary embolism and was associated with a 28-fold increased venous thromboembolism risk [9]. This travel risk is not limited to long distance air flights as a French study demonstrated that long distance car travel is also a common risk factor for venous thromboembolism [2]. Similarly lifestyle and in particular occupations are becoming increasingly sedentary [9] thereby placing populations at heightened risk. In the recent New Zealand case control study, prolonged seated immobility at work in the four weeks prior to the index event (defined as a maximum duration of sitting of ≥10 hours/day and at least >2 hours at a time without getting up) was present in 17% of patients with venous thromboembolism and was associated with a 2.8-fold increased venous thromboembolism risk [9]. Predominantly sedentary occupations, broadly grouped into professional including IT, clerical, and administration, were at a greater risk of venous thromboembolism than less sedentary occupations such as labourers, technicians, and trade workers. Thus, venous thromboembolism secondary to prolonged seated immobility in different situations, including long distance air and car travel, work, and recreation, represents a major potential burden and high priority for preventive measures. 

The subjects assessed their overall experience with the MMC as favourable, being easy to apply, and comfortable to wear, both when not in operation and while “on” with the rhythmical compressions. Subjects reported that they were particularly likely to consider wearing the device in the hospital setting and may use it during long distance travel, suggesting that the MMC provides a prophylactic option in these situations at least. Potential advantages of this novel device in the context of long distance travel are its light weight and comfort.

In conclusion, this study has confirmed that the MMC causes a marked increase in lower limb blood flow and is easy to wear and comfortable when in operation. The increase in peak systolic venous velocity and other measures suggest that the MMC may provide an attractive strategy to reduce venous thromboembolism risk in situations associated with prolonged periods of immobility, such as long distance travel. The MMC also has potential to be used as an alternative to the conventional tethered IPC systems commonly prescribed for postsurgical venous thromboembolism prevention in the hospital setting. 

## Figures and Tables

**Table 1:** Characteristics of participants.

Variable	Mean (SD)
Age (years)	35.6 (11.8)
Height (m)	1.77 (0.08)
Weight (kg)	68.6 (9.9)
BMI (kg/m^2^)	21.9 (2.5)

	*N*/*N* (%)

Sex, female	8/19 (42.10%)

BMI: body mass index.

**Table 2:** Clinical measurements with the MMC and GCS interventions, during seated immobility. Data are presented as mean (SD).

Variable	Time in minutes			
	−30	−10	30	60
	Mean (SD)			
Peak systolic velocity (cm/s)				
MMC	5.65 (1.08)	5.27 (1.01)	20.1 (6.77)	20.1 (8.05)
GCS	5.61 (1.05)	4.99 (1.02)	5.66 (1.17)	5.63 (1.13)

Mean flow velocity (cm/s)				
MMC	2.30 (0.53)	2.04 (0.47)	5.78 (1.85)	5.86 (2.08)
GCS	2.38 (0.48)	2.06 (0.41)	2.45 (0.48)	2.38 (0.44)

Total volume flow (L/min)				
MMC	0.10 (0.03)	0.10 (0.04)	0.29 (0.15)	0.30 (0.17)
GCS	0.12 (0.03)	0.11 (0.03)	0.13 (0.04)	0.12 (0.04)

Vein area (cm^2^)				
MMC	0.74 (0.20)	0.80 (0.24)	0.83 (0.24)	0.83 (0.27)
GCS	0.86 (0.24)	0.87 (0.26)	0.89 (0.26)	0.82 (0.21)

Circumference (cm)				
MMC				
Ankle	22.1 (1.6)	22.2 (1.5)	22.3 (1.5)	22.4 (1.6)
Calf	38.0 (2.5)	38.3 (2.5)	38.5 (2.5)	38.7 (2.5)
GCS				
Ankle	22.1 (1.6)	22.3 (1.8)	22.5 (1.5)	22.6 (1.7)
Calf	38.0 (2.8)	38.6 (2.5)	37.6 (2.2)	37.6 (2.2)

**Table 3:** Participant subjective feedback responses.

	Mean (SD)
Please rate your experience of the following aspects of the foot compression device (MMC)^#^:	
Ease of application	4.42 (0.69)
Comfort (when not in operation)	4.00 (0.88)
Comfort of the rhythmical compression	4.42 (0.51)
Overall experience	4.05 (0.40)
Please rate the likelihood that you would consider wearing this device to help prevent DVT in the following settings*:	
Hospital	4.42 (0.69)
Air travel	3.37 (1.30)
Seated at home	2.79 (1.23)
Seated at work	2.79 (1.23)

^#^Scoring system 1 = very poor, 2 = poor, 3 = neutral, 4 = good, 5 = very good.

*Scoring system 1 = very low, 2 = low, 3 = neutral, 4 = high, 5 = very high.
